# Default mode network functional connectivity negatively associated with trait openness to experience

**DOI:** 10.1093/scan/nsab048

**Published:** 2021-04-23

**Authors:** Maja Rou Marstrand-Joergensen, Martin K Madsen, Dea S Stenbæk, Brice Ozenne, Peter S Jensen, Vibe G Frokjaer, Gitte M Knudsen, Patrick M Fisher

**Affiliations:** Neurobiology Research Unit, Rigshospitalet, Copenhagen University Hospital, Copenhagen 2100, Denmark; Faculty of Health and Medical Sciences, University of Copenhagen, Copenhagen 2200, Denmark; Neurobiology Research Unit, Rigshospitalet, Copenhagen University Hospital, Copenhagen 2100, Denmark; Faculty of Health and Medical Sciences, University of Copenhagen, Copenhagen 2200, Denmark; Neurobiology Research Unit, Rigshospitalet, Copenhagen University Hospital, Copenhagen 2100, Denmark; Neurobiology Research Unit, Rigshospitalet, Copenhagen University Hospital, Copenhagen 2100, Denmark; Department of Public Health, Section of Biostatistics, University of Copenhagen, Copenhagen 1014, Denmark; Neurobiology Research Unit, Rigshospitalet, Copenhagen University Hospital, Copenhagen 2100, Denmark; Neurobiology Research Unit, Rigshospitalet, Copenhagen University Hospital, Copenhagen 2100, Denmark; Faculty of Health and Medical Sciences, University of Copenhagen, Copenhagen 2200, Denmark; Department of Psychiatry Copenhagen, Mental Health Services Capital Region of Denmark, Copenhagen 2100, Denmark; Neurobiology Research Unit, Rigshospitalet, Copenhagen University Hospital, Copenhagen 2100, Denmark; Faculty of Health and Medical Sciences, University of Copenhagen, Copenhagen 2200, Denmark; Neurobiology Research Unit, Rigshospitalet, Copenhagen University Hospital, Copenhagen 2100, Denmark

**Keywords:** resting-state fMRI, default mode network, trait openness, generalized least squares, personality neuroscience

## Abstract

Evaluating associations between the five-factor personality domains and resting-state functional connectivity networks (e.g. default mode network, DMN) highlights distributed neurobiological systems linked to behaviorally relevant phenotypes. Establishing these associations can highlight a potential underlying role for these neural pathways in related clinical illness and treatment response. Here, we examined associations between within- and between-network resting-state functional connectivity with functional magnetic resonance imaging and the five-factor personality domains: Openness to experience (Openness), Extraversion, Neuroticism, Agreeableness and Conscientiousness. We included data from 470 resting-state scan sessions and personality assessments in 295 healthy participants. Within- and between-network functional connectivity from 32 a priori defined regions was computed across seven resting-state networks. The association between functional connectivity and personality traits was assessed using generalized least squares. Within-network DMN functional connectivity was significantly negatively associated with trait Openness (regression coefficient = −0.0010; [95% confidence interval] = [−0.0017, −0.0003]; *P*_FWER_ = 0.033), seemingly driven by association with the Fantasy subfacet. Trait Extraversion was significantly negatively associated with functional connectivity between the visual and dorsal attention networks and positively associated with functional connectivity between the frontoparietal and language networks. Our findings provide evidence that resting-state DMN is associated with trait Openness and gives insight into personality neuroscience.

## Background

The five-factor model of personality is a widely recognized model for personality, as measured by the NEO Personality Inventory (NEO PI-R), consisting of trait Openness to Experience (Openness), Extraversion, Neuroticism, Agreeableness and Conscientiousness (Costa and McCrae, 1992). Openness is related to sensitivity to feeling, aesthetic experience and openness toward new ideas and values (McCrae and Costa 2006; Fayn *et al.*, 2015); individuals high in this trait tend to exhibit increased cognitive flexibility (Fleischhauer *et al.*, 2010; DeYoung *et al.*, 2014), creativity (Li *et al.*, 2015), intellectual curiosity and motivation for novel-seeking experiences (McCrae and John, 1992). Extraversion is associated with positive affect and describes an outgoing person (Smillie *et al.*, 2015). Neuroticism reflects a sensitivity and nervous reactivity to stressful situations (Perkins *et al.*, 2007). Agreeableness is related to compassionate and friendly behavior (Graziano *et al.*, 2007). Conscientiousness is related to self-discipline and well-organized behavior (Ozer and Benet-Martínez, 2006). Additionally, each of the five personality factors in NEO PI-R are defined by a subdivision of six facets, providing a detailed and characteristic description of the personality traits through a total of 30 facet scales (Costa and McCrae, 1995; Ekehammar and Akrami, 2007; Han and Pistole, 2017).

Resting-state functional magnetic resonance imaging (rs-fMRI) is a non-invasive brain imaging tool that can estimate so-called ‘resting-state networks’ (RSNs) and provides a framework for delineating neural pathways underlying individual variability in personality traits (Nostro *et al.*, 2018). Several RSNs have been identified and described with rs-fMRI (Biswal *et al.*, 1995; Beckmann *et al.*, 2005; Salvador *et al.*, 2005; Damoiseaux *et al.*, 2006; De Luca *et al.*, 2006; van den Heuvel *et al.*, 2008). Perhaps the most widely studied is the default mode network (DMN) (Raichle *et al.*, 2001), which includes the right and left lateral parietal, medial prefrontal and posterior cingulate cortices (Greicius *et al.*, 2003; Buckner *et al.*, 2008). These areas show correlated and increased metabolic activity when the brain is at rest or engaged in mind-wandering, compared to decreased metabolic activity when attending to a specific task or stimulus (Raichle, 2015). Resting-state fMRI studies have found that DMN is associated with a wide range of cognitive phenomena such as self-reference (Whitfield-Gabrieli *et al.*, 2011), social behavior (Xie *et al.*, 2016), rumination (Hamilton *et al.*, 2011) and emotional states (Zidda *et al.*, 2018). The DMN has been linked to creativity and imagination through increased functional connectivity with cognitive control brain systems (Beaty *et al.*, 2018) and increased activity during tasks involving social cognition (Murphy *et al.*, 2019). Altered DMN functional connectivity has been reported in several neurological (Lucas-Jimenez *et al.*, 2016; Mohan *et al.*, 2016) and psychiatric (Davey *et al.*, 2012; Sambataro *et al.*, 2014; Chen *et al.*, 2017; Zhao *et al.*, 2019) disorders. Indeed, changes in personality are linked to several brain disorders including cognitive impairment, behavioral changes, affective disorders, psychosis, irritability, delirium and chronic fatigue (Butler and Zeman, 2005), implicating a convergence of RSN dysfunction and personality.

Multiple studies have examined associations between resting-state functional connectivity and core personality traits applying different analysis methodologies (Adelstein *et al.*, 2011; Kunisato *et al.*, 2011; Dubois *et al.*, 2018; Mulders *et al.*, 2018; Nostro *et al.*, 2018; Toschi *et al.*, 2018). Recent research has associated psychoticism with Openness (DeYoung *et al.*, 2016), highlighting an association between this behaviorally relevant phenotype and neuropsychiatric illness. Openness has also been linked to DMN dynamic functional connectivity (Beaty *et al.*, 2018), increased functional connectivity putatively related to dopamine signaling between substantia nigra/ventral tegmental area and the dorsolateral prefrontal cortex (Passamonti *et al.*, 2015), functional connectivity strength between parietal regions linked to memory (Wang *et al.*, 2018), DMN efficiency (Beaty *et al.*, 2016) and DMN coherence (Blain *et al.*, 2020), and resting-state functional connectivity in DMN and dorsolateral prefrontal cortex (Adelstein *et al.*, 2011). A recent study on 365 healthy participants reported a positive, although not statistically significant, association between DMN functional connectivity and Openness measured using the 50-item International Personality Item Pool (IPIP) (Simon *et al.*, 2020). This finding would be further strengthened by a similar observation with the NEO PI-R (Costa and McCrae, 1992), which is among the most well-validated and broadly applied questionnaires for quantifying personality traits across research frameworks. Intriguingly, recent studies have reported that psilocybin (psychoactive component in ‘magic mushrooms’) both affects resting-state functional connectivity, including decreased DMN (Carhart-Harris *et al.*, 2012), and increases Openness (MacLean *et al.*, 2011; Carhart-Harris *et al.*, 2016; Madsen *et al.*, 2020).

A comprehensive neuroimaging study in 884 healthy participants from the Human Connectome Project reported that Openness alone and a combination personality trait derived from Openness, Neuroticism and Extraversion were best predicted by resting-state data (Dubois *et al.*, 2018). Another study reported an association between Openness and resting-state functional connectivity in meta-analytically defined networks associated with emotion processing such as reward and pain, and executive functions such as vigilant attention using relevance vector machine learning (Nostro *et al.*, 2018).

Together, these findings suggest a convergent link between Openness and RSNs that should be examined in additional datasets. Whether trait Openness is especially associated with DMN compared to other core personality traits using the NEO PI-R questionnaire in a large cohort of healthy, Danish subjects has not previously been evaluated. Thus, we hypothesized that within resting-state DMN functional connectivity is specifically associated with Openness.

Here, we explored the association between the five-factor personality traits and within- and between-network resting-state FC in a large cohort comprising 470 rs-fMRI scan sessions acquired in 295 unique healthy participants. Additionally, we examined associations of related facets.

## Methods

### Participants

Data included in the study were drawn from the Center for Integrated Molecular Brain Imaging (Cimbi) database (Knudsen *et al.*, 2016). All descriptive characteristics of the healthy participants were collected during the first recruitment of the participant, in cases where the participant had been recruited for more than one project. Initially, 488 rs-fMRI datasets were identified in the Cimbi database from 297 unique healthy individuals who also had a NEO PI-R assessment. Datasets were excluded if the rs-fMRI acquisition was more than 1 year (365 days) from the NEO PI-R assessment nearest in time. Our dataset included 470 rs-fMRI scan sessions from 295 unique healthy participants. Of these individuals, 147 completed a single scan session, 121 completed two scan sessions, and 27 completed three scan sessions. Data were acquired between February 2010 and September 2018.

All participants were healthy and had no past history or current neurologic or psychiatric disorders as examined by a trained clinician. All participants tested negative for drug urine screen prior to MRI scan. Exclusion criteria included a history of substance abuse, treatment with psychopharmacological drugs, significant medical disorders, head trauma, regular use of medication for any neurological/psychiatric disease or severe illnesses, non-fluency in Danish, pregnancy or breastfeeding. Written informed consent was obtained from all participants following the Helsinki Declaration. The associated studies were approved by Capital Region’s Ethics Committee: VEK (KF)01-2006-20 + appendix (KF)23830, VEK H-15003302, VEK (KF)01280377, VEK H-1-2010-085, VEK H-15017713, VEK H-3-2013-100, VEK H-2-2010-108, VEK (KF)01-2006-20, VEK H-6-2014-057, VEK H-16026898, VEK H-15004506. Some of the functional connectivity data presented here were included in a previous study (Fisher *et al.*, 2017).

### NEO personality questionnaires

All participants completed a Danish version of either the NEO Personality Inventory Revised (PI-R) (Costa and McCrae, 1992) or the updated version NEO Personality Inventory 3 (NEO PI-3) (McCrae *et al.*, 2005), each of which returns an identical factor structure. Both versions comprise 240 items and the scores from the two inventories can be aligned without adjustment (De Fruyt *et al.*, 2009). For the NEO PI-R, participants rated items on a five-point Likert rating scale from 0 (‘strongly disagree’) to 4 (‘strongly agree’). NEO-PI-3 items were rated from 1 (‘strongly disagree’) to 5 (‘strongly agree’). All participants completed the questionnaire through the Hogrefe online system (https://www.hogrefe-online.com). Personality factor and facet scores were determined by summing the scores from relevant items, loading on to respective factors and facets. Internal consistency, as measured by Cronbach’s alpha, for each personality trait was as follows: Neuroticism = 0.90, Extraversion = 0.88, Openness = 0.88, Agreeableness = 0.88 and Conscientiousness = 0.90. For participants with multiple completed NEO PI-R assessments (*n* = 148), we paired each rs-fMRI scan session with the personality assessment nearest in time to the corresponding rs-fMRI scan date.

### MRI data acquisition

MRI scan sessions were completed on one of five 3 Tesla (3T) MRI scanners: 3T Trio, 3T mMR, 3T Prisma and two 3T Verio MRI scanners. Resting-state fMRI scans were 10 minutes in length. Detailed scanner information and scanner-specific sequence parameters can be found in Supplementary Table S1. For all participants, we acquired (i) a high-resolution T1-weighted structural scan, (ii) a B0 field map to correct for B0 inhomogeneities and (iii) EPI rs-fMRI scans while participants relaxed in the scanner with eyes closed and were asked to let their mind wander and not fall asleep.

### Image analysis

Resting-state fMRI data were preprocessed using SPM12 (https://www.fil.ion.ucl.ac.uk/spm/, Wellcome Department of Cognitive Neurology, London, UK) and MATLAB R2019b (Mathworks, Natick, MA). Functional images were slice-timing corrected, field map distortion corrected and realigned to the first volume. The high-resolution, T1-weighted structural image was co-registered to the functional images; segmented into gray matter, white matter and cerebrospinal fluid; and normalized into Montreal Neurological Institute (MNI) space. Functional images were normalized into MNI space using the estimated deformations (final voxel size: 2 × 2 × 2 mm) and smoothed using an 8-mm full-width half-maximum Gaussian kernel to limit spatial variance introduced by the normalization. Additional denoising of functional images was performed in ‘Conn’ (Whitfield-Gabrieli and Nieto-Castanon, 2012), including temporal band-pass filtering (0.008–0.01 Hz) and aCompCor (Behzadi *et al.*, 2007), which estimates physiological noise sources from principal components (PCs) of white matter and cerebrospinal fluid time series (first five PCs from each). The following time series were also included for denoising: six estimated motion parameters (and first derivatives), composite framewise displacement motion estimated in Artifarct Detection Tools (ART) and by the spatial root mean square variance over voxels after temporal differencing (DVARS). (Power *et al.*, 2012) and the first derivative of the first five PCs from white matter and cerebrospinal fluid. Individual volumes with excessive Blood-Oxygen-Level-Dependent (BOLD) signal variance and head motion were censored using ART (global signal variance threshold = 4 s.d. values, composition motion > 2). No participants were excluded due to excessive motion. Data quality of anatomical and functional images was verified by visual inspection, including tissue segmentation and head motion.

For each rs-fMRI scan session, functional connectivity was estimated as the Fisher’s *r*-to-*z* transformation of the correlation coefficient (rho) between denoised regional time series for all pairs of regions defined a priori by the ‘networks’ atlas in ‘Conn’. This atlas defines eight RSNs from 32 discrete brain regions: DMN, sensorimotor network (SMN), visual network (VN), dorsal-attention network (DAN), salience network (SN), frontoparietal network (FPN), language network (LN) and cerebellar network (CN) (Supplementary Table S2). Pearson’s correlation coefficients were estimated for each region pair and transformed using Fisher’s *r*-to-*z* transformation (i.e. 0.5 × [ln(1 + r) − ln(1 − r)], where *r* is the correlation coefficient and ‘ln’ is the natural logarithm). Within- and between-network functional connectivity estimates were calculated as the mean of all *r*-to-*z* values for a specific within- or between-network (e.g. DMN within-network functional connectivity was calculated as mean of the six unique connections between the four regions within DMN; DMN–SMN between-network functional connectivity was calculated as mean of the 12 unique DMN–SMN connections). This resulted in eight within-network and 28 between-network estimates per rs-fMRI scan session.

### Statistics

Descriptive characteristics are presented as mean and s.d., median and interquartile range (IQR), or *n* and percentage, as appropriate. We assessed the association between the big five personality factors and the mean within- and between-network functional connectivity of DMN, SMN, VN, SN, DAN, FPN, LN and CN by generalized least-squares regression, using ‘corSymm’ as a covariance structure to account for the correlation between repeated measurements over the same subject (Maggin *et al.*, 2011). Median composite motion for each rs-fMRI session correlated with Openness (rho = 0.12, *P*-value = 0.04; associations with all other personality factors’ *P*-values > 0.05) and was included as a covariate with sex, age and MRI scanner. Residuals from generalized least-squares regression models were plotted in QQ plots and inspected visually for normal distribution assumption. Statistical significance estimates (i.e. *P*-values) for the associations between a personality factor and all within- and between-network functional connectivity estimates (i.e. 36 tests) were adjusted for multiple comparisons using Dunnett’s procedure (*P*_FWER_), which controls the family-wise error rate (α = 0.05) (Dmitrienko and D’Agostino, 2013). Subfacets of personality factors showing statistically significant associations with network functional connectivity were examined. This included the association between Openness and DMN and between Extraversion and VN-DAN and FPN-LN functional connectivity. To more closely examine whether personality traits were associated with functional connectivity when adjusting for the other personality factors, an additional set of analyses was carried out including all personality factors as regressors, in addition to the above-mentioned covariates. As above, the family-wise error rate on statistical significance estimates across the 36 models was controlled using Dunnett’s procedure.

All statistical analyses were two-tailed and the level of statistical significance was set to *P*-value < 0.05. All reported analyses were performed in the statistical software package R (version 3.3.456, https://cran.r-project.org/) or by SPSS Statistics (IBM Corp. Released 2016. IBM SPSS Statistics for Macintosh, Version 24.0. Armonk, NY: IBM Corp).

## Results

### Participant demographics

We included 295 healthy participants (mean ± s.d. age at first MRI scan: 26.2 ± 6.4 years; sex distribution: 54% female; Table 1). Personality questionnaires were completed near in time to the related MR scan session (median [IQR]: 0 [−5, +8] days; range: −270, +216 days relative to MR scan session; Table 1). As expected, all within-network RSNs showed general positive resting-state functional connectivity (Supplementary Figure S1). Scanner differences in within-network functional connectivity are reported in Supplementary Table S3.

**Table 1.** Descriptive characteristics of participants

	Mean ± s.d. or *n* (%)	Median (IQR)	Range, min–max
Demographics			
Age (years)	26.19 ± 6.43	24.39 (21.83–28.00)	18.41–60.08
Sex (female)	158 (53.6%)		
BMI (kg/m^2^)	23.57 ±** **3.17	23.10 (21.27–25.35)	16.89–36.85
Personality assessment			
NEO PI-R	279 (94.6%)		
NEO PI-3	16 (5.4%)		
Days from MRI scan to NEO PI-R/NEO PI-3	2.86 ± 36.82	0 (−5–8)	−270–216
Trait Neuroticism score	79.75 ± 21.48	78 (64–94)	17–139
Trait Extraversion score	121.84 ± 19.28	126 (110–135.5)	48–162
Trait Openness score	121.64 ± 19.38	121 (108–134)	68–174
Trait Agreeableness score	122.57 ± 19.03	123 (111–135)	47–174
Trait Consci-entiousness score	112.91 ± 20.96	111 (97.5–129)	42–171
MRI scanners			
3T Trio	87 (29.5%)		
3T Verio-1	62 (21.0%)		
3T mMR	13 (4.4%)		
3T Prisma	107 (36.3%)		
3T Verio-2	26 (8.8%)		

Data are presented in mean ± s.d., median (IQR), range or n (%), as appropriate.

Min: minimum. Max: maximum. BMI: Body mass index. NEO PI-R: NEO Personality Inventory revised. NEO PI-3: NEO Personality Inventory 3. All reported data were collected during the first recruitment of the participant.

### DMN functional connectivity and openness

**Fig. 1.**
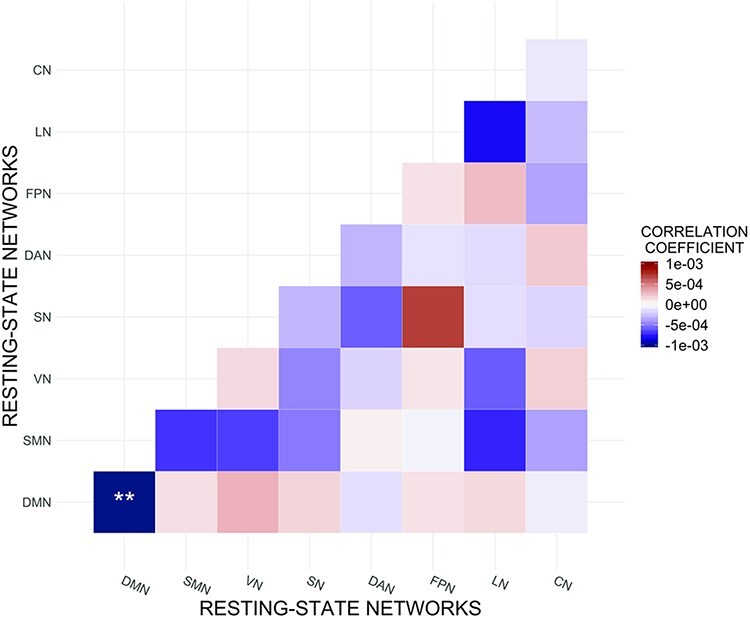
Associations between Openness and resting-state functional connectivity.

**Fig. 2.**
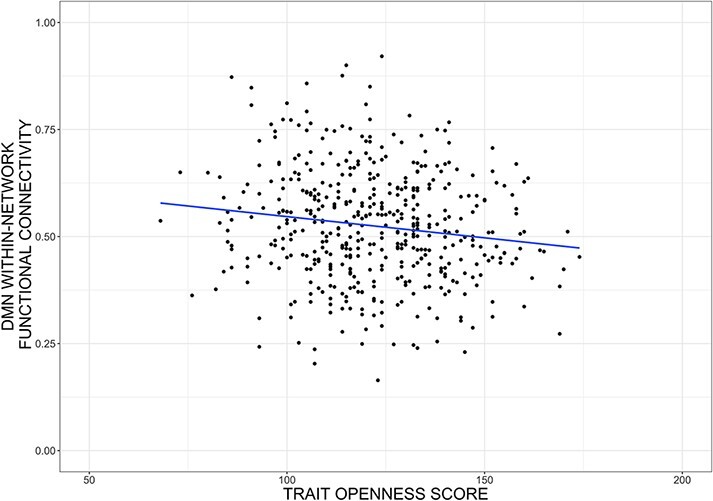
Openness association with DMN functional connectivity.

Within-network DMN functional connectivity was statistically significantly negatively associated with Openness (regression coefficient = −0.0010, [95% confidence interval (CI)] = [−0.0017, −0.0003], *P*_FWER_ = 0.033; Table 2, Figures 1 and 2). Upon adding Extraversion, Neuroticism, Agreeableness and Conscientiousness as additional regressors to the regression model, the association between DMN and Openness remained negative (−0.0008; [−0.0015, −0.00003]; *P*_FWER_ = 0.22). *Post hoc* analyses between Openness facets and DMN functional connectivity identified Fantasy as the only facet showing a statistically significantly negative correlation (−0.0036, [−0.0059, −0.0011], *P*_FWER_ = 0.031; Supplementary Table S4). 

**Table 2.** Associations between Openness and within-network functional connectivity

Networks	Regression coefficient	SE	95% CI	Standardized regression coefficient	*P*-value	*P*_FWER_
DMN	−0.0010	0.0004	−0.0017, −0.0003	−0.15	0.004	0.033
SMN	−0.0006	0.0005	−0.0017, 0.0003	−0.064	0.22	0.63
VN	0.0002	0.0005	−0.0009, 0.0012	0.018	0.72	0.99
SN	−0.0003	0.0004	−0.0010, 0.0004	−0.041	0.43	0.88
DAN	−0.0003	0.0005	−0.0013, 0.0007	−0.034	0.51	0.92
FPN	0.0001	0.0005	−0.0009, 0.0011	0.013	0.80	0.99
LN	−0.0007	0.0004	−0.0016, −0.00002	−0.091	0.080	0.34
CN	−0.0001	0.0004	−0.0010, 0.0008	−0.0066	0.89	1.0

Parameters from generalized least-squares model, data comprise 470 resting-state fMRI scans from 295 unique individuals. Model covariates: age, sex, MRI scanner and median composite motion. SE: Standard Error. *P*-values corrected for 36 within- and between-network tests using multiplicity adjustment by Dunnett’s procedure (*P*_FWER_).

Heatmap showing associations between Openness and respective within- and between-network resting-state functional connectivity estimates. Red indicates positive associations whereas blue indicates negative associations, shaded by magnitude of association. Values indicate the correlation coefficient from respective generalized least-squares regression models including age, sex, MRI scanner and median composite motion as covariates. ** denotes *P*_FWER_ < 0.05 after adjustment with Dunnett’s procedure.

Openness is negatively associated with DMN functional connectivity. Black dots indicate individual observed data points. Blue line indicates regression line from generalized least-squares regression model. Functional connectivity values were obtained by adjusting for the effect of the model covariates age, sex, MRI scanner and median composite motion on functional connectivity estimated in the generalized least-squares regression model.

### Between-network functional connectivity and extraversion

**Fig. 3.**
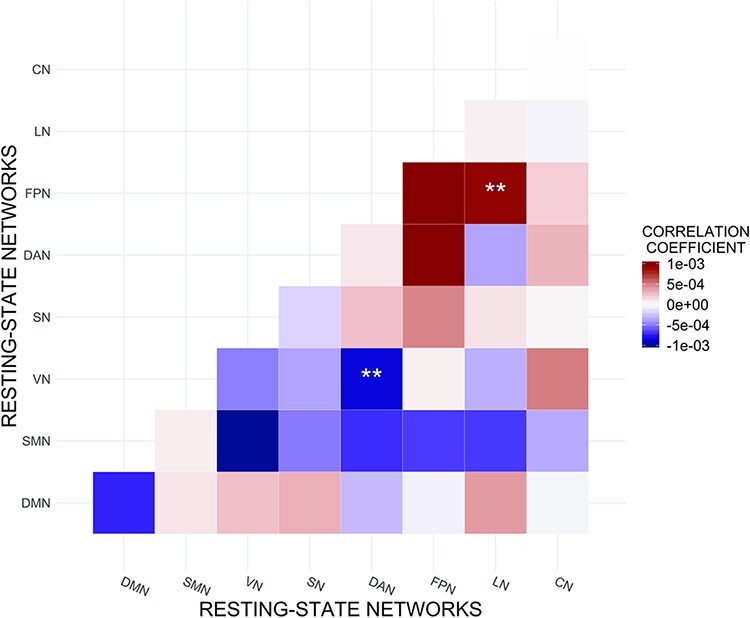
Associations between Extraversion and resting-state functional connectivity.

**Fig. 4.**
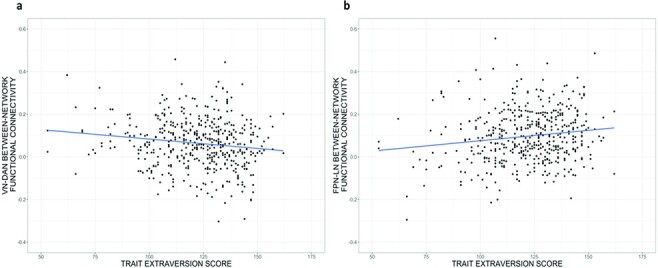
Extraversion associated with between-network functional connectivity.

Between-network functional connectivity of VN–DAN was statistically significantly negatively associated with Extraversion (−0.0009; [−0.0011, −0.0001]; *P*_FWER_ = 0.031) and FPN–LN between-network functional connectivity was statistically significantly positively associated with Extraversion (0.0009; [0.0003, 0.0016]; *P*_FWER_ = 0.027, Table 3, Figures 3 and 4). Upon adding Openness, Neuroticism, Agreeableness and Conscientiousness as additional regressors to the regression model, the association with VN–DAN remained negatively associated with Extraversion (−0.001 [−0.0017, −0.0003]; *P*_FWER_ = 0.048, whereas the association with FPN–LN remained positive (0.0008 [0.00006, 0.0015]; *P*_FWER_ = 0.17). *Post hoc* facet-level analysis of Extraversion indicated that Warmth was significantly negatively associated with VN–DAN (−0.004; [−0.0068, −0.0012]; *P*_FWER_ = 0.038, Supplementary Table S4) and Positive Emotions was significantly positively associated with FPN–LN (0.0039; [0.0014, 0.0064]; *P*_FWER_ = 0.015, Supplementary Table S4). No other facets of Extraversion were significantly associated with between-network functional connectivity of VN-DAN or FPN-LN (*P*_FWER_ > 0.05). 

**Table 3.** Associations between Extraversion and between-network functional connectivity

Networks	Regression coefficient	SE	95% CI	Standardized regression coefficient	*P*-value	*P*_FWER_
DMN–SMN	0.0001	0.0003	−0.0005, 0.0007	0.016	0.73	0.99
DMN–VN	0.0003	0.0003	−0.0003, 0.0008	0.046	0.38	0.80
DMN–SN	0.0003	0.0003	−0.0003, 0.0009	0.050	0.33	0.75
DMN–DAN	−0.0003	0.0003	−0.0009, 0.0003	−0.052	0.31	0.73
DMN–FPN	−0.0001	0.0004	−0.0008, 0.0006	−0.0087	0.86	0.99
DMN–LN	0.0004	0.0004	−0.0003, 0.0011	0.057	0.27	0.67
DMN–CN	−0.00004	0.0003	−0.0007, 0.0006	−0.0066	0.90	1.0
SMN–VN	−0.0010	0.0004	−0.0017, −0.0001	−0.12	0.02	0.13
SMN–SN	−0.0005	0.0003	−0.0012, −0.00004	−0.084	0.10	0.37
SMN–DAN	−0.0007	0.0004	−0.0015, 0.0001	−0.090	0.07	0.28
SMN–FPN	−0.0007	0.0004	−0.0014, −0.00003	−0.088	0.06	0.26
SMN–LN	−0.0007	0.0003	−0.0011, 0.00002	−0.12	0.02	0.13
SMN–CN	−0.0004	0.0003	−0.0010, 0.0003	−0.055	0.28	0.69
VN–SN	−0.0004	0.0003	−0.0010, 0.0003	−0.070	0.16	0.51
VN–DAN	−0.0009	0.0003	−0.0011, −0.0001	−0.15	0.0045	0.031
VN–FPN	0.0001	0.0003	−0.0005, 0.0006	0.013	0.80	1.0
VN–LN	−0.0003	0.0003	−0.0009, 0.0002	−0.058	0.23	0.62
VN–CN	0.0005	0.0004	−0.00004, 0.0013	0.072	0.17	0.51
SN–DAN	0.0003	0.0003	−0.0003, 0.0008	0.047	0.34	0.76
SN–FPN	0.0005	0.0004	−0.0002, 0.0012	0.072	0.18	0.53
SN–LN	0.0001	0.0004	−0.0006, 0.0008	0.018	0.73	0.99
SN–CN	0.00004	0.0003	−0.0006, 0.0006	0.0076	0.88	1.0
DAN–FPN	0.0006	0.0003	0.00001, 0.0012	0.096	0.05	0.24
DAN–LN	−0.0004	0.0003	−0.0010, 0.0001	−0.068	0.18	0.53
DAN–CN	0.0003	0.0003	−0.0003, 0.0009	0.050	0.32	0.74
FPN–LN	0.0009	0.0003	0.0003, 0.0016	0.14	0.0036	0.027
FPN–CN	0.0002	0.0004	−0.0005, 0.0009	0.027	0.59	0.96
LN–CN	−0.0001	0.0003	−0.0005, 0.0005	−0.0086	0.85	1.0

Parameters from generalized least-squares model, data comprise 470 resting-state fMRI scans from 295 unique individuals. Model covariates: age, sex, MRI scanner and median composite motion. *P*-values corrected for 36 within- and between-network tests using multiplicity adjustment by Dunnett’s procedure (*P*_FWER_).

Heatmap showing associations between Extraversion and respective within- and between-network resting-state functional connectivity estimates. Red indicates positive associations whereas blue indicates negative associations, shaded by magnitude of association. Values indicate the correlation coefficient from respective generalized least-squares regression models including age, sex, MRI scanner and median composite motion as covariates. ** denotes *P*_FWER_ < 0.05 after adjustment with Dunnett’s procedure.

(a) Extraversion negatively associated with VN–DAN functional connectivity, (b) Extraversion positively associated with FPN–LN functional connectivity. Black dots indicate individual observed data points. Blue line indicates regression line from generalized least-squares regression model. Functional connectivity values were obtained by adjusting for the effect of the model covariates age, sex, MRI scanner and median composite motion on functional connectivity estimated in the generalized least-squares regression model.

Beyond effects reported above, no other associations between personality factors and within- or between-network resting-state functional connectivity were statistically significant (*P*_FWER_ > 0.07 for all).

## Discussion

In the current study, we examined the associations between core five-factor personality traits and resting-state functional connectivity in a large cohort of healthy individuals. We observed that resting-state DMN functional connectivity was negatively associated with Openness, including the Fantasy facet. Extraversion was significantly negatively associated with VN–DAN between-network functional connectivity and significantly positively associated with FPN–LN between-network functional connectivity. The Extraversion facet Warmth was negatively associated with VN–DAN between-network functional connectivity whereas the facet Positive Emotions was positively associated with FPN–LN between-network functional connectivity. No other personality factors were statistically significantly associated with any within- or between-network resting-state functional connectivity estimates, besides from the associations with Openness and Extraversion. Our findings support an association between Openness and the widely studied DMN. More broadly, the limited number of significant associations, including the absence of an association with three core personality traits, indicates a limited association between canonical RSNs and core personality traits.

We observed a significant negative association between Openness and within DMN functional connectivity, the only statistically significant association for a within-network functional connectivity estimate. A recent rs-fMRI study including 365 healthy participants reported a positive, although not statistically significant, association between Openness and DMN functional connectivity (Simon *et al.*, 2020). Simon and colleagues also reported a negative association between trait Neuroticism and DAN functional connectivity and negative associations between trait Neuroticism and trait Agreeableness and the ventral attention network. However, discrepancies in data analysis are notable. The previous study estimated Openness from the 50-item IPIP and DMN functional connectivity from the 58-region set described previously (Power *et al.*, 2011). Our scan sessions were longer (10 minutes *vs* 5 or 9.5 minutes), which may affect connectivity variance. Simon and colleagues excluded negative correlation values when computing DMN functional connectivity, stemming from uncertainty about how to interpret negative correlations (Simon *et al.*, 2020). It is our preference to include all available data in part because data censoring raises issues of generalizability. Negative functional connectivity has previously been studied and validated (Gopinath *et al.*, 2015; Keller *et al.*, 2015; Delaveau *et al.*, 2017; Parente *et al.*, 2018). Negative functional connectivity can emerge from, e.g. global signal regression (Murphy *et al.*, 2009; Weissenbacher *et al.*, 2009), but studies also report the presence of negative correlations in the absence of global signal regression (Fox *et al.*, 2009; Chai *et al.*, 2012). Interestingly, we did not perform global signal regression during denoising. Nevertheless, only 2% of our DMN functional connectivity values were <0, suggesting the inclusion/exclusion of negative connectivity values has a limited effect on the discrepancy of our findings. Thus, although our findings are not directionally aligned, direct comparison of the results is not straightforward due to the above-mentioned methodological differences.

Recent smaller rs-fMRI studies investigating the association between Openness and RSNs have applied data-driven independent component analysis (ICA) (Wang *et al.*, 2018), graph theory measures (Beaty *et al.*, 2016) or dynamic functional connectivity (Beaty *et al.*, 2018). A recent dynamic functional connectivity rs-fMRI study showed that trait Openness/Intellect is significantly associated with increased dwell time in a brain state including connections between DMN regions and cognitive control regions (Beaty *et al.*, 2018). We examined the association between static functional connectivity and Openness and found no associations with between-network functional connectivity estimates. The discrepancy between the findings of the current study and the study by Beaty *etal.* is not surprising since two different methodological approaches for measuring the rs-fMRI have been used. Dynamic functional connectivity estimated ‘brain states’ reflect a composite of regions across networks and dwell time (Beaty *et al.*, 2018) and is not necessarily comparable to static functional connectivity within a well-defined RSN such as DMN. Similarly, a recent study applied graph theory and reported that Openness was positively correlated with global efficiency estimated within a 34-region definition of the DMN (Beaty *et al.*, 2016). Graph measures reflect an estimate of capacity for information flow, e.g. network efficiency (Latora and Marchiori, 2001) and information processing (Achard and Bullmore 2007), but are not directly relatable to an association between DMN functional connectivity and Openness. Thus, Openness may be both positively related to DMN efficiency and negatively associated with DMN functional connectivity. Although we fully agree that data-driven and more complex analytic strategies are useful and informative, we view our current approach as advantageous in offering a transparent functional connectivity metric and association with Openness that is easily interpretable.

The negative association between DMN functional connectivity and Openness is convergent with serotonin psychedelic studies reporting decreased resting-state functional connectivity with DMN regions (Carhart-Harris *et al.*, 2012; Smigielski *et al.*, 2019) and increased Openness (MacLean *et al.*, 2011; Erritzoe *et al.*, 2019; Madsen *et al.*, 2020). The above-mentioned studies are consistent with a model wherein Openness is negatively associated with DMN and we speculate that environmental factors such as serotonin psychedelics may translate an individual’s position along the axis of this association. Future neuroimaging studies before and after the administration of psychedelics could more concretely establish whether lasting psychedelic-induced increases in Openness are accompanied by corresponding lasting negative effects on DMN functional connectivity.

Intriguingly, recent studies suggest a link between Openness and psychoticism, a characteristic of psychotic-like symptoms but without severe schizophrenic illness (Kwapil *et al.*, 2008; DeYoung *et al.*, 2016). A recent study reported that Openness and psychoticism were associated with increased DMN coherence, as measured by the average correlation between voxels defined to belong to DMN as defined by an ICA component (Blain *et al.*, 2020). Although our finding is not aligned with this study, this may stem from methodological and quantification differences. Notably, two other studies have reported a negative association between psychotic-like experiences and dynamic DMN functional connectivity and DMN global efficiency (Sheffield *et al.*, 2016; Barber *et al.*, 2018).

We observed that Extraversion was statistically significantly associated with two between-network estimates, namely a negative association with VN-DAN and a positive association with FPN-LN functional connectivity. We are not aware of a previous study directly evaluating the between-network effect that we observed but our finding suggests between-network functional connectivity should be considered in future personality neuroimaging studies. People with high Extraversion scores tend to be outgoing (Fishman *et al.*, 2011), show positive affect (Smillie *et al.*, 2015) and are motivated by reward pursuit (Smillie *et al.*, 2019). A previous study using dynamic functional connectivity found that patients with major depressive disorder had a significantly shorter dwell time in a brain state constituted by strong functional connections between SMN, auditory network, VN and DMN compared to healthy controls (Wu *et al.*, 2019). The dwell time in this brain state was positively correlated with Extraversion and negatively correlated with Neuroticism. In our study, we report that Extraversion is negatively associated with VN–DAN functional connectivity and positively associated with FPN–LN functional connectivity. Thus, some of the dynamic functional connectivity associated with Extraversion reported by Wu and colleagues could be related to static functional connectivity between RSNs.

*Post hoc* analyses of personality facets indicated facets of Openness and Extraversion were associated with resting-state functional connectivity. The Openness facet Fantasy, which describes persons with an imaginative mind (Ekehammar and Akrami, 2007; Han and Pistole, 2017) was negatively associated with DMN functional connectivity. The Extraversion facet Warmth, which describes tenderness and kindness (Ekehammar and Akrami, 2007; Han and Pistole, 2017), was negatively associated with VN-DAN functional connectivity. The Extraversion facet Positive Emotions, which reflects an overall feeling of well-being (Ekehammar and Akrami, 2007; Han and Pistole, 2017), was positively associated with FPN–LN between-network functional connectivity. Future studies considering the association between personality traits and RSNs should consider whether they can replicate these effects before drawing stronger inference of these associations.

We observed only three statistically significant associations between resting-state functional connectivity and any of the five-factor personality traits examined; we did not observe a significant association for three of the personality traits, i.e. Agreeableness, Conscientiousness and Neuroticism. Previous studies have reported a similar scale of associations (Beaty *et al.*, 2016; Mulders *et al.*, 2018; Toschi *et al.*, 2018; Simon *et al.*, 2020), which nevertheless highlight the likely limitation of rs-fMRI in explaining neurobiological mechanisms underlying personality. However, Nostro *etal.* was able to predict four out of five personality factors using relevance vector machine learning (Nostro *et al.*, 2018), but Dubois *etal*. was only able to significantly predict Openness (Dubois *et al.*, 2018) and reported that only ∼2% of the variance in Openness was predicted by whole-brain rs-fMRI analyses. Taken together, these findings indicate that there is substantial work to be done in delineating the neurobiological mechanisms associated with core personality traits and alternatives to rs-fMRI should be actively pursued.

Certain limitations should be taken into consideration when interpreting our reported results. Although we considered a large dataset of 470 rs-fMRI scans from 295 unique individuals, data acquisition was carried out across five different 3T MRI scanners, adding heterogeneity to our dataset. We attempted to model this variability by including MRI scanner as a covariate in all regression models. If anything, we expect scanner heterogeneity would decrease our ability to detect statistically significant associations with Openness. The participants in our sample have significantly higher Openness scores compared to a reported Danish norm sample (Skovdahl *et al.*, 2011) and are generally very healthy, as defined by exclusion criteria. Although this may limit generalizability of our findings, Figure 2 does not obviously suggest a non-linear relation as Openness decreases to the normative range. However, there are relatively few data points with which to draw a firm conclusion and we note this potential limitation.

In conclusion, we observed a negative association between DMN functional connectivity and Openness and its facet Fantasy. Extraversion was significantly negatively associated with VN–DAN between-network functional connectivity and significantly positively associated with FPN–LN between-network functional connectivity. The Extraversion facet Warmth was negatively associated with VN–DAN functional connectivity, whereas the facet Positive Emotions was positively associated with FPN–LN functional connectivity. Openness was not significantly associated with any other network functional connectivity estimate, nor were any other five-factor personality measures. These findings reinforce the relevance of DMN functional connectivity as a neurobiological correlate of a core personality trait.

## Supplementary Material

nsab048_Supp
