# Supplementary material for: Default mode network functional connectivity negatively associated with trait openness to experience
Source: Soc Cogn Affect Neurosci. 2021 Apr 23;16(9):950–61. doi: 10.1093/scan/nsab048 (PMC8610093; doi:10.1093/scan/nsab048)
Supplement: nsab048_Supp [file nsab048_supp.zip › Supplementary_Material_Table_S1.docx]

**Supplementary Material Table S1.**

| **MRI scanner** | **Acq. time** | **Vol.** | **TR** | **TE** | **Flip angle** | **Slice thickness** | **Slices** | **Distance factor** | **Acquisition order** | **FOV** | **Voxel size** |
| --- | --- | --- | --- | --- | --- | --- | --- | --- | --- | --- | --- |
| **3T**  **Trio** | 10.08 | 244 | 2490 | 30 | 20° | 3 | 42 | 0% | interleaved | 192 | 3.0 x 3.0 x 3.0 |
| **3T**  **Verio-1** | 10.02 | 280 | 2150 | 26 | 78° | 3 | 42 | 0% | interleaved | 192 | 3.0 x 3.0 x 3.0 |
| **3T**  **Verio-2** | 10.02 | 280 | 2150 | 26 | 78 | 3 | 42 | 0% | interleaved | 192 | 3.0 x 3.0 x 3.0 |
| **3T mMR** | 10.02 | 280 | 2150 | 26 | 78° | 3 | 42 | 0% | interleaved | 192 | 3.0 x 3.0 x 3.0 |
| **3T**  **Prisma** | 10.0 | 300 | 2000 | 30 | 90 | 3 | 32 | 25% | interleaved | 230 | 3.6 x 3.6 x 3.0 |
