# Supplementary material for: Default mode network functional connectivity negatively associated with trait openness to experience
Source: Soc Cogn Affect Neurosci. 2021 Apr 23;16(9):950–61. doi: 10.1093/scan/nsab048 (PMC8610093; doi:10.1093/scan/nsab048)
Supplement: nsab048_Supp [file nsab048_supp.zip › Supplementary_Material_Table_S2.docx]

**Supplementary Material Table S2.**

| **Default mode network** | **MNI coordinates** |
| --- | --- |
| Medial prefrontal cortex | (1,55,-3) |
| Posterior cingulate cortex | (1,-61,38) |
| Left lateral parietal cortex | (-39,-77,33) |
| Right lateral parietal cortex | (47,-67,29) |
| **Sensori-Motor network** | |
| Sensori Motor lateral left | (-55,-12,29) |
| Sensori Motor lateral right | (56,-10,29) |
| Sensori Motor superior | (0,-31,67) |
| **Visual network** | |
| Visual Primary | (2,-79,12) |
| Visual Ventral | (0,-93,-4) |
| Visual Dorsal left | (-37,-79,10) |
| Visual Dorsal right | (38,-72,13) |
| **Salience network** | |
| Anterior cingulate cortex | (0,22,35) |
| Anterior insula left | (-44,13,1) |
| Anterior insula right | (47,14,0) |
| Rostrale prefrontal cortex left | (-32,45,27) |
| Rostrale prefrontal cortex right | (32,46,27) |
| Supramarginal gyrus left | (-60,-39,31) |
| Supramarginal gyrus right | (62,-35,32) |
| **Dorsal-Attention network** | |
| Frontal eye fields left | (-27,-9,64) |
| Frontal eye fields right | (30,-6,64) |
| Intraparietal sulcus left | (-39,-43,52) |
| Intraparietal sulcus right | (39,-42,54) |
| **Fronto-Parietal network** |  |
| Lateral prefrontal cortex left | (-43,33,28) |
| Lateral prefrontal cortex right | (41,38,30) |
| Posterior parietal cortex left | (-46,-58,49) |
| Posterior parietal cortex right | (52,-52,45) |
| **Language network** | |
| Inferior frontal gyrus left | (-51,26,2) |
| Inferior frontal gyrus right | (54,28,1) |
| Posterior superior temporal gyrus left | (-57,-47,15) |
| Posterior superior temporal gyrus right | (59,-42,13) |
| **Cerebellar network** | |
| Cerebellar anterior | (0,-63,-30) |
| Cerebellar posterior | (0,-79,-32) |
