# Supplementary material for: Default mode network functional connectivity negatively associated with trait openness to experience
Source: Soc Cogn Affect Neurosci. 2021 Apr 23;16(9):950–61. doi: 10.1093/scan/nsab048 (PMC8610093; doi:10.1093/scan/nsab048)
Supplement: nsab048_Supp [file nsab048_supp.zip › Supplementary_Material_Table_S3.docx]

**Supplementary Material Table S3.**

| **Networks** | **3T Trio** | **3T Verio-1** | **3T mMR** | **3T Prisma** | **3T Verio-2** | **P-value** |
| --- | --- | --- | --- | --- | --- | --- |
| **DMN** | 0.49 ± 0.14 | 0.47 ± 0.12 | 0.53 ± 0.20 | 0.48 ± 0.13 | 0.45 ± 0.13 | 0.39 |
| **SMN** | 0.56 ± 0.18 | 0.52 ± 0.19 | 0.62 ± 0.19 | 0.62 ± 0.21 | 0.53 ± 0.21 | 0.014 |
| **VN** | 0.57 ± 0.15 | 0.68 ± 0.18 | 0.58 ± 0.16 | 0.49 ± 0.20 | 0.57 ± 0.18 | 2x10e-8 |
| **SN** | 0.43 ± 0.14 | 0.49 ± 0.14 | 0.55 ± 0.08 | 0.56 ± 0.14 | 0.50 ± 0.12 | 5x10e-7 |
| **DAN** | 0.36 ± 0.17 | 0.39 ± 0.17 | 0.53 ± 0.23 | 0.50 ± 0.18 | 0.45 ± 0.20 | 3x10e-6 |
| **FPN** | 0.49 ± 0.19 | 0.55 ± 0.17 | 0.64 ± 0.15 | 0.69 ± 0.18 | 0.58 ± 0.16 | 9x10e-11 |
| **LN** | 0.49 ± 0.14 | 0.51 ± 0.13 | 0.53 ± 0.14 | 0.60 ± 0.16 | 0.58 ± 0.14 | 2x10e-4 |
| **CN** | 0.59 ± 0.18 | 0.58 ± 0.15 | 0.57 ± 0.20 | 0.75 ± 0.17 | 0.74 ± 0.16 | 2x10e-11 |
