# Supplementary material for: Default mode network functional connectivity negatively associated with trait openness to experience
Source: Soc Cogn Affect Neurosci. 2021 Apr 23;16(9):950–61. doi: 10.1093/scan/nsab048 (PMC8610093; doi:10.1093/scan/nsab048)
Supplement: nsab048_Supp [file nsab048_supp.zip › Supplementary_Material_Table_S4.docx]

**Supplementary Material Table S4.**

|  | **Regression coefficient** | **Standardized regression coefficient** | ***SE*** | **95% CI** | **P-value** | **P_FWER_** |
| --- | --- | --- | --- | --- | --- | --- |
| **DMN associations with Openness facets** | | | | | | |
| *Fantasy* | -0.0036 | -0.018 | 0.0012 | -0.0059, -0.0011 | 0.004 | **0.031** |
| *Aesthetics* | -0.0014 | -0.0087 | 0.0011 | -0.0035, 0.0008 | 0.21 | 0.64 |
| *Feelings* | -0.0028 | -0.013 | 0.0015 | -0.0056, 0.0003 | 0.07 | 0.31 |
| *Actions* | -0.0041 | -0.016 | 0.0018 | -0.0076, -0.0009 | 0.02 | 0.11 |
| *Ideas* | -0.0026 | -0.014 | 0.0012 | -0.0050, -0.0002 | 0.04 | 0.19 |
| *Values* | -0.0013 | -0.004 | 0.0021 | -0.0056, 0.0024 | 0.54 | 0.93 |
| **VN-DAN associations with Extraversion facets** | | | | | | |
| *Warmth* | -0.004 | -0.15 | 0.0014 | -0.0068; -0.0012 | 0.005 | **0.038** |
| *Gregariousness* | -0.0026 | -0.11 | 0.0012 | -0.0049; -0.00026 | 0.03 | 0.15 |
| *Assertiveness* | -0.0011 | -0.046 | 0.0012 | -0.0034; 0.0012 | 0.36 | 0.84 |
| *Activity* | -0.0026 | -0.097 | 0.0014 | -0.0053; 0.00009 | 0.06 | 0.25 |
| *Excitement seeking* | -0.0035 | -0.13 | 0.0014 | -0.0062; -0.0008 | 0.01 | 0.07 |
| *Positive Emotions* | -0.002 | -0.087 | 0.0012 | -0.0044; 0.0003 | 0.09 | 0.36 |
| **FPN-LN associations with Extraversion facets** | | | | | | |
| *Warmth* | 0.0039 | 0.13 | 0.0015 | 0.0010; 0.0069 | 0.008 | 0.06 |
| *Gregariousness* | 0.0022 | 0.089 | 0.0012 | -0.0003; 0.0045 | 0.08 | 0.33 |
| *Assertiveness* | 0.0018 | 0.070 | 0.0012 | -0.0007; 0.0042 | 0.15 | 0.53 |
| *Activity* | 0.0037 | 0.13 | 0.0014 | 0.0009; 0.0065 | 0.01 | 0.06 |
| *Excitement seeking* | 0.0009 | 0.03 | 0.0015 | -0.0020; 0.0037 | 0.55 | 0.93 |
| *Positive Emotions* | 0.0039 | 0.16 | 0.0013 | 0.0014; 0.0064 | 0.002 | **0.015** |
