# Supplementary figures and images for: Default mode network functional connectivity negatively associated with trait openness to experience
Source: Soc Cogn Affect Neurosci. 2021 Apr 23;16(9):950–61. doi: 10.1093/scan/nsab048 (PMC8610093; doi:10.1093/scan/nsab048)

**Supplementary Material Figure S1.**

**
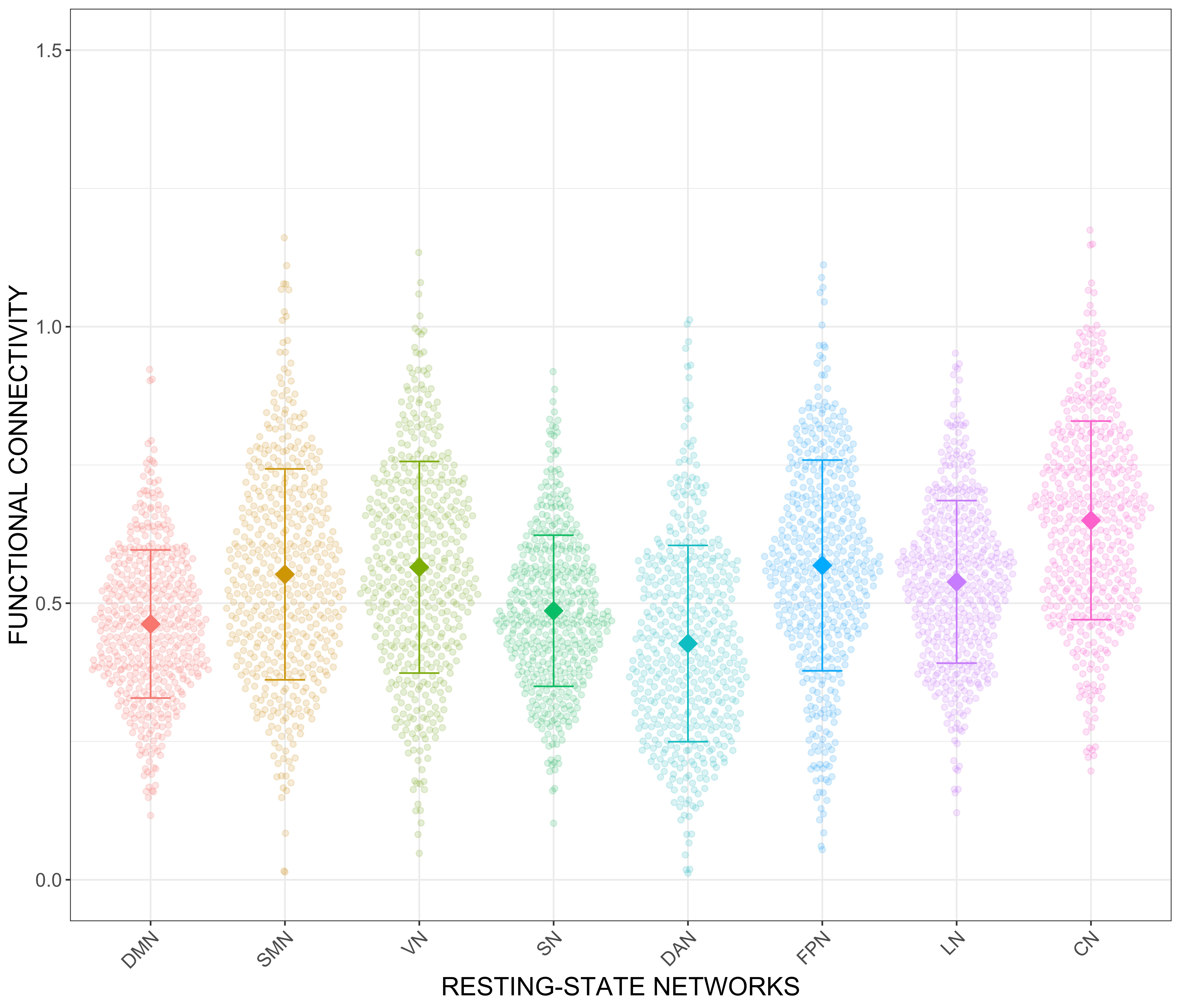
**

Supplement: nsab048_Supp [file nsab048_supp.zip › Supplementary_Material_Figure_S1.docx]
